# Explicit and implicit monitoring in neurodegeneration and stroke

**DOI:** 10.1038/s41598-019-50599-x

**Published:** 2019-10-01

**Authors:** Indira Garcia-Cordero, Lucas Sedeño, Andrés Babino, Martín Dottori, Margherita Melloni, Miguel Martorell Caro, Mariano Sigman, Eduar Herrera, Facundo Manes, Adolfo M. García, Agustín Ibáñez

**Affiliations:** 1 Institute of Cognitive and Translational Neuroscience (INCYT), INECO Foundation, Favaloro University, Buenos Aires, Argentina; 2 National Scientific and Technical Research Council (CONICET), Buenos Aires, Argentina; 3 Departamento de Física J.J. Giambiagi, FCEyN - UBA and IFIBA-CONICET, Pabellón 1, Ciudad Universitaria, Buenos Aires, 1428 Argentina; 4 Laboratory of integrative neuroscience, The Rockefeller University, New York, NY 10065 USA; 5 Torcuato Di Tella University, Buenos Aires, Argentina; 6 Facultad de Lenguas y Educación, Universidad Nebrija, Madrid, Spain; 7 Departamento de Estudios Psicológicos Universidad ICESI, Cali, Colombia; 8 Centre of Excellence in Cognition and its Disorders, Australian Research Council (ARC), Sydney, Australia; 9 Faculty of Education, National University of Cuyo (UNCuyo), Mendoza, Argentina; 10 Center for Social and Cognitive Neuroscience (CSCN), School of Psychology, Universidad Adolfo Ibáñez, Santiago de Chile, Chile; 11 Universidad Autónoma del Caribe, Calle 90, Barranquilla, Colombia

**Keywords:** Cognitive ageing, Cognitive neuroscience

## Abstract

Monitoring is a complex multidimensional neurocognitive phenomenon. Patients with fronto-insular stroke (FIS), behavioural variant frontotemporal dementia (bvFTD) and Alzheimer’s disease (AD) show a lack of self-awareness, insight, and self-monitoring, which translate into anosognosia and daily behavioural impairments. Notably, they also present damage in key monitoring areas. While neuroscientific research on this domain has accrued in recent years, no previous study has compared monitoring performance across these brain diseases and none has applied a multiple lesion model approach combined with neuroimaging analysis. Here, we evaluated explicit and implicit monitoring in patients with focal stoke (FIS) and two types of dementia (bvFTD and AD) presenting damage in key monitoring areas. Participants performed a visual perception task and provided two types of report: *confidence* (explicit judgment of trust about their performance) and *wagering* (implicit reports which consisted in betting on their accuracy in the perceptual task). Then, damaged areas were analyzed via structural MRI to identify associations with potential behavioral deficits. In AD, inadequate confidence judgments were accompanied by poor wagering performance, demonstrating explicit and implicit monitoring impairments. By contrast, disorders of implicit monitoring in FIS and bvFTD patients occurred in the context of accurate confidence reports, suggesting a reduced ability to turn self-knowledge into appropriate wagering conducts. MRI analysis showed that ventromedial compromise was related to overconfidence, whereas fronto-temporo-insular damage was associated with excessive wagering. Therefore, joint assessment of explicit and implicit monitoring could favor a better differentiation of neurological profiles (frontal damage vs AD) and eventually contribute to delineating clinical interventions.

## Introduction

Monitoring is a complex multidimensional neurocognitive phenomenon. The capacity to reflect on our own cognitive processes and change behavior accordingly^1–4^ encompasses both explicit and implicit dimensions^5^. The former can be tapped through self-report of confidence on task-specific behavioral outcomes^2,6^, while the latter can be assessed considering the subjects’ wagers on how well they performed^3,7^. However, the literature on such distinctions is ambiguous, as monitoring has been proposed as a global widespread phenomenon linked to frontal lobe function^8^ or as a domain-specific process relying on circumscribed brain areas^9,10^. Moreover, patients with neurological conditions present several daily living difficulties mainly related with poor monitoring of cognitive and behavioural decline^5,11,12^. The study of this phenomenon is particularly important for clinical interventions, as a proper understanding of monitoring dysfunctions could reduce treatment drop-out and maximize therapeutic effectiveness^13^. Against this background, the present study aims to characterize monitoring processes through the evaluation of lesion models in patients with neurodegeneration and stroke.

One key determinant of monitoring is the ability to consciously track one’s own behavior during task performance, a capacity linked to the ventromedial prefrontal cortex (vmPFC). This skill, which proves critical for achieving self-control and guiding conduct^2,6^, can be tapped through explicit self-reports of confidence on behavioural outcomes^2,6^. However, monitoring does not exclusively rely on explicit operations, as it also involves implicit processing of information^5^. This can be assessed considering the subjects’ wagers on how well they performed^3,7^, as these hinge on implicit knowledge to maximize earnings. Notably, although implicit processing has been related with the integrity of the fronto-temporo-insular hubs^14,15^, no study has tested the role of this network in scenarios which require tacit integration processes for accurate implementation of knowledge^5^. In this sense, given that explicit and implicit monitoring processes are necessary for accurate self-awareness, a dissociation between them could produce different patterns of anosognosia (unawareness of a deficit or disease) and behavioural impairments^5^. Therefore, the neural basis of monitoring deficits needs to be deeply tackled.

This gap can be bridged through the lesion model approach, which reveals direct links between behavioural performance and compromised brain regions^16,17^. This framework allows detecting critical substrates of specific functions by studying the association between behavioral deficits and the location of brain damage (being focal or diffuse). This approach proves more informative when behavioral examinations are combined with neuroimaging techniques (in our case, structural MRI), which allows for a multilevel assessment of how a brain regional compromise impacts in particular neurocognitive processes^18^, highlighting their pathophysiological mechanisms^19,20^. Here, we focused on patients with fronto-insular stroke (FIS) and two neurodegenerative conditions (behavioural variant frontotemporal dementia [bvFTD] and early stage Alzheimer’s disease [AD]). Patients from these three conditions systematically show lack of self-awareness, insight, and self-monitoring^19–22^, which translates into anosognosia^19^ and daily behavioural impairments^19^. The combined study of FIS, bvFTD, and AD^23–26^ may reveal critical brain regions related to both dimensions of monitoring. In particular, FIS patients provide a convergent lesion model to assess critical regions underlying monitoring at large, given that focal damage proves robust than diffuse atrophy models for establishing anatomo-clinical correlations^16,17^. The bvFTD and AD groups provide a contrastive lesion (neurodegenerative) model to explore areas which are commonly affected in neurodegenerative diseases relevant to monitoring dimensions. Notably, patents with such conditions also present damage in key monitoring areas, thus affording suitable lesion models to explore explicit and implicit mechanisms. In short, the combination of multiple lesion models, such as stroke and neurodegeneration^24,25^, offers unique opportunities to better characterize the brain correlates of explicit and implicit monitoring.

Against this background, this work aimed to reveal the critical brain areas involved in explicit and implicit monitoring by applying multimodal lesion models combined with structural neuroimaging. Based on previous studies, and considering that all these pathologies involve reduced self-awareness, we predicted that both explicit and implicit monitoring would be impaired in our three patient groups, with worse performance on implicit measures. Neuroanatomically speaking, we hypothesized that the vmPFC would be mainly associated with explicit monitoring, while fronto-temporo-insular regions would be related to implicit post-decision wagering. All in all, this study seeks to explore the neural basis of distinct monitoring dimensions by revealing their differential disruptions in neuropathological models.

## Materials and Methods

### Participants

The study comprised 75 participants, belonging to four groups: FIS (*n* = 18), bvFTD patients (*n* = 21), AD patients (*n* = 16), and healthy controls (*n* = 20). Fronto-insular stroke patients presented non-hemorrhagic, fronto-insular lesions provoked by stroke. They were evaluated at least six months post-stroke to ensure lesion stability and presentation of post-acute clinical symptomatology. Diagnosis of probable bvFTD was made following current revised criteria^27^. Patients in this group were in early/mild stages of the disease and presented social and behavioral impairments, as defined by caregivers^15,28,29^. Moreover, they exhibited fronto-temporo-insular atrophy on MRI and frontal hypoperfusion in SPECT recordings, when available. Alzheimer’s disease patients were diagnosed in accordance with the NINCDS-ADRDA criteria^30,31^. Diagnoses of both neurodegenerative conditions were established by expert clinicians and supported by an extensive neurological, neuropsychiatric, and neuropsychological examination, as in previous reports^24,29,32,33^. None of the patients gave signs of other forms of dementia. Neither did they fulfil criteria for specific psychiatric disorders. The control group was matched in age, education, and gender with all patient groups and had no history of psychiatric or neurological disease. All participants provided signed informed consent in accordance with the Declaration of Helsinki. All methods were implemented in accordance with the relevant institutional guidelines and regulations. The study was approved by the Ethics Committee of the Institute of Cognitive Neurology. See Table 1 for participants’ demographic information.

**Table 1:** Participants’ demographic data.

	FIS patients	bvFTD patients	AD patients	Controls	Statistics	*p*-values	*p*-values post-hoc^d^
Male: female	8/10	12/9	3/13	8/12	FIS^b^: *x*^2^ (1): 0.07 bvFTD^b^: *x*^2^ (1): 1.20 AD^b^: *x*^2^ (1): 1.89	NS NS NS	
Age: mean (SD) Range^a^	62.00 (7.06) 52–76	69.81 (9.97) 40–84	74.19 (8.46) 50–83	68.05 (7.61) 54-80	F(3,71)^c^: 6.22	<.001	FIS: NS bvFTD: NS AD: NS
Education: mean (SD) Range^a^	13.00 (3.45) 3–17	14.38 (4.53) 5–24	12.63 (4.53) 6–24	15.10 (3.32) 8–18	F(3,71)^c^: 1.55	NS	

^a^In years.

^b^Gender: Chi-squared test against controls.

^c^One-way ANOVA between groups.

^d^Dunnet test against controls.

NS: non significant.

### Experimental task

The task used in this study was adapted from a classic non-verbal paradigm used to measure monitoring abilities in humans (adults and children)^34^ as well as non-human primates^35^. The task comprised 114 trials, each starting with a perceptual judgment followed by a report of confidence in performance. Confidence was measured, through reports of *confidence* (explicit monitoring) or *wagering* (implicit monitoring). In the perceptual task, subjects had to identify the largest circle in a screen of nine black circles of different sizes. The difficulty of this task varied from easy to hard trials, adjusted by the size of the largest circle relative to the rest. Ninety-four follow-up trials measured monitoring processes, as participants had to report, after their judgment, how confident they were about their choice. In half of these trials (47), confidence was reported on a continuous colour slider, ranging from red (indicating low confidence) to blue (indicating higher confidence). This yielded an explicit monitoring measure which we succinctly refer to as *confidence*, calculated as the mean of the values selected in the slider. In the other 47 trials, subjects were asked to earn as many points as possible by making a binary choice between a yellow button which involved a wager in their response or a violet button one which implied to opt-out and go for a safe but more modest payoff. Specifically, if the yellow button was pressed following a correct response in the perceptual task, participants earned three points (shown as red balls on the side of the screen). Instead, if that button was pressed after an incorrect response, they lost three points. Finally, if the violet button was pressed, participants earned one point, irrespective of their performance in the perceptual task (see Fig. 1.1). These binary trials yielded an implicit monitoring measure, named *wagering*, calculated as the number of times the subjects pressed the yellow button divided by the number of total wagering-type trials. The rest of the trials consisted in 20 plain trials (with no confidence report). Trials were presented randomly. In addition, we included a fraction of very easy trials, in which participants were expected to achieve close-to-perfect performance, which were used as catch trials. Participants received explicit instructions about the scoring system and they were told that their goal was to earn as many points as they could. Before the experiment, they were asked to complete five practice trials for task-familiarization purposes.

**Figure 1:**
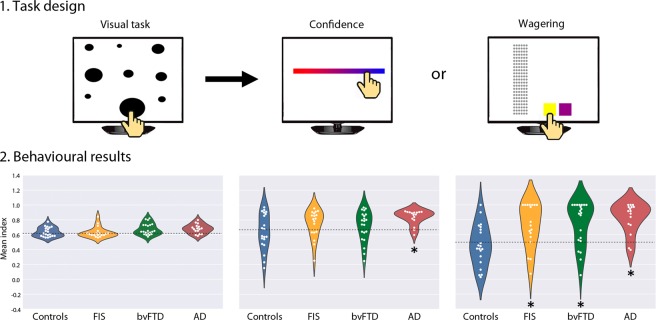
Task design and behavioral results. 1. Task design. The perceptual task consists in the selection of the largest circle of the screen. The follow-up task involves a monitoring report (confidence or wagering) based on the perceptual task (left panel). In the middle panel, the slider indicates continuous values of confidence, from low (red) to high (blue). The right panel shows the wagering screen: the yellow button should be selected if the participant is sure of the election of the circle (three points are earned in correct responses or subtracted in incorrect responses); the violet one button should be pressed when the participant is not sure about his previous selection (adding one point). Earned points are displayed on the side of the screen. 2. Groups’ performance in the tasks. The left panel shows the matched first-order performance between all groups. The middle panel shows significant overconfidence for AD patients compared to controls. The right panel shows that all patient groups significantly differed from controls in their wagering performance. The asterisk (*) indicates significant differences relative to controls. Dotted lines indicate the mean index for controls. FIS: fronto-insular stroke, bvFTD: behavioral variant frontotemporal dementia, AD: Alzheimer’s disease.

Since first-order performance could influence monitoring^4,36^, we ensured that all participants performed similarly in the visual task. Difficulty was controlled through a QUEST procedure^37^, which uses the past responses and the Bayes rule (plus random noise) to compute the 75%-performance threshold of the next step. To maximize reliability of these baseline data, we discarded for the analysis the first 25 trials (from the total 114 trials). In addition, visual task performance (calculated as the number of correct answers in all trials divided by the total number of trials) was matched between groups and outliers were eliminated considering two standard deviations below and above the mean performance of controls^38^ (see results section 3.1 and Fig. 1.2 left). The visual task was considered as a baseline first-order condition about which the participants reported their confidence or wagered. Therefore, no brain correlates were analyzed from this performance.

As the mean confidence and mean wagering values were considered individual representative and reliable measures of confidence^39^, both measures for each patient group were compared about those of controls through one-way ANOVAs and Dunnet post-hoc tests.

### MRI acquisition and analysis

MRI acquisition and preprocessing steps are reported in accordance with the practical guidelines from the Organization for Human Brain Mapping^40^. A few participants presented excessive movements during acquisition and were excluded from analysis. The final samples for MRI analyses consisted of 15 FIS patients, 20 bvFTD patients, 15 AD patients, and 19 controls. Demographic data for each these reduced patient samples were also matched with those of the control group. See more details in supplementary file 1.2 and Supplementary Table 1.

#### Lesion analysis

In agreement with previous studies^24–26^, lesion masks for the FIS patients were manually traced in native patient spaces according to visible damage on T1 and T2 scans. All masks were normalized to MNI space and then overlapped to obtain the lesion map (Fig. 2.1).

**Figure 2:**
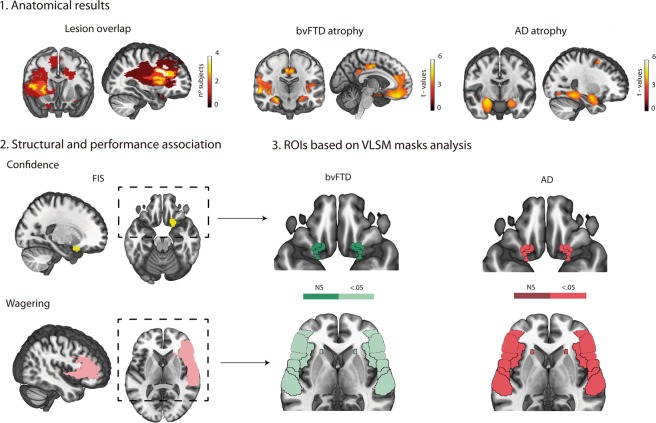
Anatomical results and structural-behavioral association. 1. Lesion overlap in FIS patients, and atrophy of bvFTD and AD patients (VBM) compared to controls (*p* < 0.001, extent threshold = 50 voxels). 2. Structures associated with confidence (upper row) and wagering (lower row) in FIS patients (VLSM, left side). Lesions in ventromedial and fronto-temporo-insular regions were related with deficits in confidence and wagering, respectively (*p* < 0.05, FDR-corrected). For bvFTD (middle side), only the GM volume from fronto-temporo-insular areas was negatively correlated with wagering (*p* < 0.05, FDR-corrected). For AD (right side), GM volumes from ventromedial and fronto-temporo-insular regions were significantly correlated with the confidence and wagering indexes (*p* < 0.05, FDR-corrected). FIS: fronto-insular stroke, bvFTD: behavioral variant of the frontotemporal dementia, AD: Alzheimer’s disease, VBM: voxel-based morphometry, VLSM: voxel-lesion symptom mapping, GM: grey matter, NS: non significant.

To infer which brain regions were critically associated with a particular cognitive deficit we relied on the voxel-based lesion-symptom mapping (VLSM) method^41,42^. This technique involves running a voxel-wise analysis, and comparing the behavioural performance of each patient with a lesion in that voxel against the ones without any lesion^41,42^. This allowed us to identify clusters of voxels that were significantly related to confidence and wagering performance. Corresponding statistical analyses were performed via the non-parametric mapping (NPM) software package^41^. We included all voxels in which at least 5% of the patients had a lesion, and the data was permutated 4000 times, with each permutation resulting in a calculated cut-off *t*-value with α = 0.05^43^. The distribution of those *t*-statistics was used to determine the cut-off score at *p* < 0.05^43^. Then, the results were corrected for multiple comparisons using the false discovery rate (FDR) method, with a *p* < 0.05.

#### Voxel-based morphometry: preprocessing and statistical analysis

Global brain atrophy patterns in bvFTD and AD patients were established via voxel-based morphometry (VBM). Data were preprocessed on the DARTEL Toolbox following validated procedures^25,26^ on Statistical Parametric Mapping software (SPM12, http://www.fil.ion.ucl.ac.uk/spm/software/spm12/).

Atrophy patterns in the bvFTD and the AD groups were calculated on SPM12 via two-sample *t*-test between each patient sample and controls (*p* < 0.001 uncorrected^25,26,44^, extent threshold = 50 voxels). For more details, see supplementary file 1.3.

#### ROI analysis

Given that focal lesions are more robust than diffuse atrophy models for establishing anatomo-clinical correlations^16,17^, and considering that monitoring processes have been linked to a diversity of regions^4^, we decided to perform a restricted analysis to the areas obtained from the VLSM analysis of FIS patients. The aim of this procedure was to explore whether the task-critical areas damaged in FIS patients were also associated with performance in bvFTD and AD. Nevertheless, and being aware that FIS-VLSM might introduce an anatomical bias to that analysis, in a second analysis we built two extended ROIs to explore other task-relevant regions possibly affected by neurodegeneration beyond the areas affected in the FIS group. This allowed us to assess the regions related with confidence and wagering beyond the results obtained from the focal lesion model (VLSM in FIS patients) and to examine possible differential effects of neurodegeneration on behaviour.

*ROIs based on the VLSM masks*: we partitioned the resulting VLSM masks of confidence and wagering into different regions, based on the Automated Anatomical Labelling (AAL) atlas^45^. Then, we extracted the GM volume from each region to explore its association with behavioural performance. Although the VLSM analysis yielded right-sided areas only, we performed this procedure in both hemispheres (mirroring the mask on the left side) to avoid right-lesion-bias from FIS patients^9,46^. We performed Pearson correlations between the GM volume in each mask and confidence and wagering scores. As done in previous reports^44,47,48^, we combined patients and controls in the correlation analysis to increase variance in scores and thus enhance statistical power to identify behavioural correlations. The analysis was corrected for multiple comparisons via FDR, with *p* < 0.05.

*Extended ROIs*: for confidence, we considered the results obtained from the VLSM analysis and added ventromedial frontal areas implicated in cognitive control, monitoring, introspection, and confidence, namely: the cingulate cortex^49,50^ and the superior and medial frontal gyri –Brodmann area 10^2,9,10,51^. For wagering, the mask included fronto-temporo-insular areas associated with implicit processes^15^. As in the previous analysis, given the bilateral contributions of the additional areas to the targeted processes^9,46^, the masks were built to cover both hemispheres and the analyses considered the GM volume from the whole mask. We performed Pearson correlations between the GM volume from each mask and behavioural performance, jointly considering patients and controls to increase statistical power^44,47,48^.

## Results

### Behavioural results

As first-order performance could influence monitoring^4,36^, accuracy of the visual task was matched between groups [*F* (3,71) = 2.49, *p* = n.s.]. See Fig. 1.2, left; and Supplementary Table 2 for details.

For explicit confidence report, AD patients showed greater levels of average confidence than controls. Instead, average confidence for FIS and bvFTD patients did not present significant differences compared to controls [F(3,71): 2.89, *p* = 0.04, ηp2 = 0.11; post- hoc comparisons against controls: FIS: *p* = n.s., bvFTD: *p* = n.s., and AD: *p* = 0.01] See Fig. 1.2, centre. The measures of wagering showed greatest sensitivity to distinguish each patients’ sample from controls, as the wagering index was significantly higher in all patient groups compared to controls [F (3,71): 6,59, *p* < 0.001, ηp2 = 0.22; post- hoc comparisons against controls: FIS: *p* = 0.01, bvFTD: *p* = 0.003, AD: *p* < 0.001]. See Fig. 1.2, right.

Finally, to examine whether the participants understood the task, we compared confidence and wagering reports for the catch trials (those with very low levels of difficulty in the perceptual task, given that the largest circle was extremely different compared to the others). We found no significant differences between patients and controls [confidence: *F*(3, 71) = 1.09, *p* = n.s.; wagering: *F*(3, 71) = 0.02, *p* = n.s.], confirming that in control trials all groups showed close to ceiling levels of confidence.

### Brain imaging results

#### Lesion overlap and atrophy pattern

The lesion overlap across FIS patients revealed predominant damage to the frontal cortices, right temporal lobe, and the right insula. The atrophy pattern in bvFTD patients affected fronto-temporo-insular regions, including the medial, middle, and orbital frontal gyri, the right fusiform gyrus, the right hippocampus, the superior and middle temporal gyri, the cingulate cortex, and portions of the parietal lobe^26^. Atrophied areas in AD patients comprised the bilateral fusiform gyrus, the right amygdala, the left hippocampus, and portions of the frontal lobe^26^. See Fig. 2.1 and Supplementary Table 3 for more details.

#### Behavioural mapping of monitoring in FIS

The VLSM analysis showed that in FIS patients, ventromedial damage was associated with higher confidence (*t*-score > 2.88, *p* < 0.05, FRD-corrected), and that fronto-temporo-insular injuries were related with excessive wagering (*t*-score > 1.82, *p* < 0.05, FRD-corrected) (see Fig. 2.2).

#### Behavioural correlates of monitoring in neurodegeneration

*ROIs based on the VLSM masks*: for confidence, only AD patients showed a (negative) association between behavioural performance and GM volume from each partitioned mask. This result indicates that AD patients with less GM volume in ventromedial areas were overconfident in the explicit monitoring task. No significant associations were found in bvFTD patients. As regards the wagering index, both bvFTD and AD patients exhibited a (negative) association between behavioural performances and GM volume from left and right fronto-temporo-insular ROIs. Therefore, the greater the atrophy of these areas, the higher the wagering index (all *p*s < 0.05, FDR-corrected). For details, see Fig. 2.3 and Supplementary Table 4.

**Figure 3:**
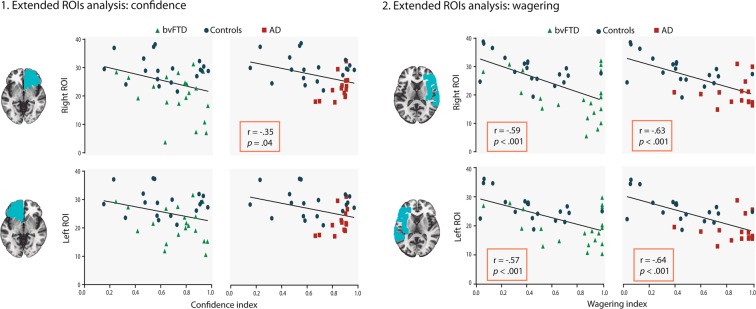
Extended ROI correlations. 1. The GM volume from the right ROI presented a significant negative correlation with confidence for AD patients and no significant correlations emerged in the bvFTD group. 2. The GM volume of the left and right ROIs negatively correlated with wagering performances in both bvFTD and AD patients. bvFTD: behavioral variant of the frontotemporal dementia, AD: Alzheimer’s disease, GM: grey matter, ROI: region of interest.

*Extended ROIs*: the GM volume in ventromedial regions was negatively associated with the confidence index for AD patients (right ROI: r = −0.35, *p* = 0.04; left ROI: r = −0.33, *p* = 0.05), but not for bvFTD (right ROI: r = −0.30, *p* = n.s.; left ROI: r = −0.29, *p* = n.s.). This indicates that the integrity of ventromedial regions was associated with good confidence reports. Wagering results were analyzed in relation to the extended fronto-temporo-insular ROI. Behavioral performances were negatively associated with GM volume in both bvFTD (right ROI: r = −0.59, *p* < 0.001; left ROI: r = −0.57, *p* < 0.001) and AD patients (right ROI: r = −0.63, *p* < 0.001; left ROI: r = −0.64, *p* < 0.001). Thus, patients with atrophy in these areas presented higher wagering performances. For details, see Fig. 3.

## Discussion

This is the first study assessing explicit and implicit monitoring based on a multiple lesion model approach. Explicit monitoring was preserved in frontal pathologies (FIS and bvFTD) but affected in AD, and it was associated with grey matter alterations of specific areas, such as the vmPFC. Implicit monitoring was altered in all conditions and it was mainly related to gray matter volume on fronto-temporo-insular regions. These results suggest a partial double dissociation between explicit and implicit processes, which seem to differentially rely on vmPFC and fronto-temporo-insular regions, respectively.

In comparison to frontal pathologies and controls, AD patients overestimated their confidence (explicit monitoring), and this pattern was related to atrophy in the vmPFC. Monitoring is closely related with executive functions and higher-order modulations of the prefrontal cortex^4^, via processes such as suppression and inhibition of information and attentional focus^5,52^. In particular, disruptions of executive processes related with vmPFC damage would induce impaired executive control and concomitant impulsiveness, disinhibition, inflexibility, poor planning, automatic processing, and perseverative behaviors^4,52,53^. Moreover, monitoring deficits have been linked to self-referential and episodic memory impairments and anosognosia in AD^12,54^. Such processes have also been related to fronto-hippocampal disconnections^20^ and, specially, to monitoring and self-awareness deficits following vmPFC lesions^20–22^. In line with these findings, our results support current models of metacognition^4,20,52,55^ in which the vmPFC receives signals from posterior regions and updates active information, facilitating the construction of new cognitive schemas to guide behaviour via top-down control processes^4,20,52,55^. This highlights the role of hippocampal-vmPFC connections in the integration of new information within the active scheme^20^. Our results confirmed that in AD, vmPFC integrity would be critical for the convergence of posterior signals in the deployment of monitoring processes.

Contrarily to that, in the frontal patients (FIS and bvFTD) explicit monitoring was preserved. In fact, this process seems to rely on fronto-posterior networks^4,20,52,55^. FIS patients presented an association between the GM volume of the vmPFC and confidence, but less posterior damage than AD patients. Although previous studies have proposed a direct role of the frontal cortex in confidence judgments, they either lacked specificity in their anatomical description^4^ or actually reported patients with vmPFC damage and spared monitoring^51^. Therefore, posterior nodes from this network might be relevant to support explicit monitoring in FIS patients. On the other hand, bvFTD patients showed a non-significant correlation between vmPFC volume and behavioural performance, suggesting that accurate confidence reports were not related with atrophy in this area. Moreover, patients with frontal damage typically perform well in office-based tasks (i.e., when the experimental setting is explicit and structured)^56,57^. However, as argued in discussions of the “frontal lobe mystery”^56,57^, office-based assessments may not be good indicators of performance in ecological, real-world settings. Therefore, the monitoring performance of the frontal patients (FIS and bvFTD) may have benefited from the structured conditions of the experiment, although this pattern may not accurately reflect their behaviour in daily life.

Regarding implicit monitoring, the three patient groups presented higher wagering scores than controls. In AD, inadequate confidence judgments were accompanied by poor wagering performance, demonstrating explicit and implicit monitoring impairments. In contrast, impairments of implicit monitoring in FIS and bvFTD patients occurred in the context of accurate confidence reports, suggesting the already proposed reduced ability to turn their self-knowledge into appropriate wagering conducts^58^. This deficit of integration of information becomes specifically evident in ecological and implicit tasks that involve everyday life problems^14,15,56^. In our implicit task, frontal patients could not use their explicit knowledge to correctly wager about the performance. Moreover, this impairment was associated with fronto-temporo-insular compromise. Indeed, similar disorders in implicit integration processes have been reported in bvFTD^14,15^ and other neuropsychiatric disorders^59,60^, following compromise of the fronto-temporo-insular hubs^15^.

This abnormal integration of implicit information could actually underlie other impairments in frontal groups (FIS and bvFTD), such as anosognosia. In particular, these patients may explicitly report their condition but fail to evaluate the behavioural, functional or cognitive consequences of their disease, directly affecting their daily living activities^5,61^. Indeed, anosognosia is the result of impaired integration processes, not only within each explicit or implicit dimension, but also between them, yielding differential patterns of monitoring affectation^5^. Therefore, the study of explicit and implicit monitoring could be proposed as an experimental proxy to assess anosognosia. In this approach, anosognosia would not restricted to explicit reports of confidence, but it also may include implicit mechanisms underlying monitoring processes. Moreover, our results are in line with the reported association between monitoring deficits and patients’ cluster of symptoms^13^. In this sense, anosognosia has been proposed to manifest in two distinct forms: (i) as cognitive denial, related with severe cognitive impairments; and (ii) as behavioural denial, correlated with worse emotional performance and disinhibition^13^. Both of these domains of anosognosia could be analogous to the two aspects of monitoring assessed herein. Explicit monitoring deficits might be related with cognitive denial, as AD patients suffer severe cognitive (especially memory^30^) decline and present failures in reporting their confidence. On the other hand, frontal patients (FIS and bvFTD) present more behavioural symptoms, such as disinhibition and social cognition deficits^27,62,63^, arguably reflecting implicit monitoring disturbances –here manifested as excessive wagering. These results highlight the relation between the patients’ perception of their disease and symptoms, which has a direct impact on the therapies they receive and the continuity of treatment^13^.

In line with this background, the social context network model (SCNM) assumes that social behaviors depends on the integration of internal and external monitoring^14,15,64^. This network operates mainly via the interaction of three key hubs: (a) an insular hub, which coordinates internal information (interoception); (b) a temporal hub, which consolidates context-target associative learning; and (c) a frontal hub, which updates external contextual cues and uses them to predict outcomes. In our experiment, both AD and bvFTD patients presented impaired implicit monitoring and an association between performance and fronto-temporo-insular grey matter. In fact, the compromise of frontal hub could disrupt executive control mechanisms, leading to disinhibition, perseveration, and disturbed emotional regulation^20,52,53^. Atrophy in the insula has been associated with social cognition impairments^65–67^ and interoceptive deficits in these groups^26^, highlighting the role of visceral feedback for implicit emotional processing^66,68–71^. In addition, associative learning indexed by temporal hubs underlies confidence judgments^11,19^. In brief, damages in these hubs may lead to impairments in the integration of implicit cues, connected with internal motivational states and episodic memories to predict future actions^14,15^. Thus, this approach provides a potential explanation for a common underlying mechanism between inhibition, social behavior, and loss of insight –symptoms markedly present across dementia patients^64^.

Finally, failures in executive functions tend to appear as a global dysfunction but, actually, they involve heterogeneous and variable impairments^72,73^. Thus, it is not suitable to estimate executive dysfunctions using a uniform approach^56^. In addition, office-based tests provide a structured environment that reduces complex demands, allowing patients to perform well in this type of assessments despite their daily life impairments. In our explicit monitoring task (a controlled and structured setting), patients with frontal damage presented an appropriate confidence report. However, these participants were not able to turn their self-knowledge into a suitable wagering performance. Patients are more sensitive to distractibility, and in a stimulus-rich context (as in the wagering task), their executive functions are more likely to fail^15^. This results support the idea that classical explicit assessments, on their own, are not enough to evaluate and differentiate FIS/bvFTD from AD^5^, given that neither explicit nor implicit measures yielded good accuracy rates when taken in isolation. In this sense, both tasks might provide a suitable way to explore monitoring profiles in dementia and focal lesions, showing greater sensitivity to detect impairments.

## Limitations

Our study presents a number of limitations, which pave the way for future research. First, our sample size was relatively small; however, it was similar to those in previous reports in stroke and dementia^25,26^. In addition, we combined three neurological conditions, individually reviewed in a multidisciplinary clinical consensus necessary for accurate diagnosis. Moreover, we strictly controlled key demographic factors. Second, we were unable to assess performance in executive function tasks, which could influence monitoring control^52^. Nevertheless, we ensured that all participants showed similar confidence levels when uncertainty was low, demonstrating that they understood the task (see results 3.1); and controlled for the first-order performance. Metacognition is closely related with the explicit dimension of monitoring process. However, monitoring emerges from an interaction between explicit and implicit processes, supported by putative neurocognitive regions and networks^5^. In addition, there is no systematic comparison among monitoring and metacognitive measures. Future studies should use the lesion model approach to assess both explicit and implicit domains and strive to unify measures in order to make the results comparable between studies.

## Conclusions

In line with neurological-based theoretical accounts^5^, our results suggest that monitoring is a complex domain with partially independent explicit and implicit dimensions. This overall finding evidenced a distinctively association of vmPFC and fronto-temporo-insular regions in the lesion models with explicit and implicit processes, respectively. In addition, the conjoint use of explicit and implicit tasks may contribute to a better differentiation of dementia profiles (frontal damage vs AD) that could impact on clinical interventions and treatments. Our work also presents a novel approach to understand the explicit and implicit monitoring and how they can be mapped into the pathological brain.

## Supplementary information

Supplementary files

## Data Availability

The datasets generated during and/or analyzed during the current study are available from the corresponding author on reasonable request.
