# Supplementary material for: Explicit and implicit monitoring in neurodegeneration and stroke
Source: Sci Rep. 2019 Oct 1;9:14032. doi: 10.1038/s41598-019-50599-x (PMC6773765; doi:10.1038/s41598-019-50599-x)
Supplement: Supplementary file 1 — Supplementary files [file 41598_2019_50599_MOESM1_ESM.pdf]

SUPPLEMENTARY FILES

Explicit and implicit monitoring in neurodegeneration and stroke

Garcia-Cordero Indira[1,2], Sedeño Lucas[1,2], Babino Andrés[3,4], Dottori Martín[1,2], Melloni Margherita[1,2], Martorell Caro Miguel[1], Sigman Mariano[5,6], Herrera Eduar[7] , Manes Facundo[1,2,8], García Adolfo M.[1,2,9], Ibáñez Agustín[1,2,8,10,11*].

[1] Institute of Cognitive and Translational Neuroscience (INCYT), INECO Foundation, Favaloro University, Buenos Aires, Argentina
[2] National Scientific and Technical Research Council (CONICET), Buenos Aires, Argentina
[3] Departamento de Física J.J. Giambiagi, FCEyN - UBA and IFIBA-CONICET, Pabellón 1, Ciudad Universitaria, Buenos Aires 1428, Argentina
[4] Laboratory of integrative neuroscience, The Rockefeller University, New York, NY, 10065, USA
[5] Torcuato Di Tella University, Buenos Aires, Argentina
[6] Facultad de Lenguas y Educación, Universidad Nebrija, Madrid, Spain
[7] Departamento de Estudios Psicológicos Universidad ICESI, Cali, Colombia
[8] Centre of Excellence in Cognition and its Disorders, Australian Research Council (ARC), Sydney, Australia
[9] Faculty of Education, National University of Cuyo (UNCuyo), Mendoza, Argentina
[10] Center for Social and Cognitive Neuroscience (CSCN), School of Psychology, Universidad Adolfo Ibáñez, Santiago de Chile, Chile
[11] Universidad Autónoma del Caribe, Calle 90, Barranquilla, Colombia

1. MATERIAL AND METHODS

1.1. MRI acquisition and analysis

We acquired whole-brain T1-weighted anatomical 3D scans, with spin echo volumes, parallel to the plane connecting the anterior and posterior commissures in a 1.5-T Phillips Intera scanner with a standard head coil (8 channels). We applied the following parameters: repetition time (TR) = 7497 ms; echo time (TE) = 3420 ms; flip angle = 8º; 256 slices; matrix dimension = 175 x 256; voxel size = 1 x 1 x 1 mm^3; sequence. T2 sequences were acquired to improve lesion/atrophy detection in each group (parameters: TR = 4863 ms; TE = 1 ms; flip angle = 8º; 22 slices, matrix dimension = 256 x 256; voxel size = 1 x 1 x 6 mm^3).

1.2. Voxel-based morphometry: preprocessing and statistical analysis

T1-weighted images in native space were first segmented using the default parameters of the SPM12 (bias regularization was set to 0.001 and bias FWHM was set to 60-mm cut-off) into white matter (WM), grey matter (GM), and cerebrospinal fluid (CFS). Then, a template was generated with the 'DARTEL (create template)' module to increase the accuracy of inter-subject alignment[10] from the complete data set using the GM and WM segmented images (default parameters indicated by SPM12). Next, we ran the 'Normalize to MNI space' module from DARTEL Tools to affine register the last template from the previous step and all GM segmented scans into MNI space.

Subsequently, all images were modulated to correct volume changes by Jacobian determinants, and avoid a bias in the intensity of an area due to its expansion during warping. Finally, in line with previous recommendations[11], an isotropic Gaussian kernel of 12-mm full width at half maximum was applied to all images. These final maps were entered in the second-level analysis to perform the correspondent statistic.

Atrophy patterns in the bvFTD and the AD groups were calculated on SPM12 via two-sample *t*-test between each patient sample and controls. Total intracranial volume was calculated by adding the native space maps of WM, GM, and CFS and was used as a covariate to discard the influence of brain-size differences ($p < .001$ uncorrected[12-14], extent threshold = 50 voxels).

Supplementary Table 1. Demographic data from the samples included in imaging analyses.

**Imaging sample**	**FIS**	**bvFTD**	**AD**	**Controls**	**Statistics**	***p values***	***p*-values post-hoc[d]**
N<br>(men/female)	15<br>(7/8)	20<br>(11/9)	15<br>(2/13)	19<br>(8/11)	FIS[b]: x^2 (1): 0.07<br>bvFTD[b]: x^2 (1): 0.65<br>AD[b]: x^2 (1): 3.34	.79<br>.42<br>.07	
Age: mean<br>(SD)<br>Range[a]	61.87<br>(6.66)<br>52-76	69.10<br>(9.68)<br>40-83	74.00<br>(8.73)<br>50-83	67.84<br>(7.76)<br>54-80	F(3,65)[c]: 5.36	.002	FIS: .11<br>bvFTD: .94<br>AD: .10
Education: mean<br>(SD)<br>Range[a]	12.87<br>(3.62)<br>3-17	14.15<br>(4.52)<br>5-24	11.87<br>(3.48)<br>6-17	15.16<br>(3.40)<br>8-18	F(3,65)[c]: 2.40	.08	

[a]in years
[b]Gender: Chi-squared test against controls
[c]One-way ANOVA between groups
[d]Dunnet test against controls
NS: Non significant

2. RESULTS

Supplementary Table 2. Behavioral indexes and statistics.

**Index**	**FIS**	**bvFTD**	**AD**	**Controls**
Confidence: mean (SD)<br>Wagering: mean (SD)<br>Performance: mean (SD)	0.75 (0.19)<br>0.73 (0.30)<br>0.63 (0.07)	0.72 (0.20)<br>0.75 (0.30)<br>0.67 (0.08)	0.84 (0.10)<br>0.83 (0.20)<br>0.68 (0.06)	0.65 (0.24)<br>0.46 (0.27)<br>0.64 (0.06)
**Statistics**	***p*-values [a]**			
Confidence<br>$F(3,71) = 2.89$, $p = .04$<br>ηp2 = 0.11	.26	.52	.01	-
Wagering<br>$F(3,71) = 6.59$, $p<.001$<br>ηp2 = 0.22	.01	.003	<.001	-
Performance<br>F(3,71) = 2.49, $p = 0.07$<br>ηp2 = 0.10	-	-	-	-

[a]Post-hoc Dunnet test against controls
NS: Non significant

Supplementary Table 3. Atrophied regions for bvFTD and AD patients relative to controls.

bvFTD atrophy						
Voxels per cluster	Regions	Coordinates			Peak t	Peak z
		x	y	Z		
1129	Angular gyrus R	30	-63	43.5	6.83	5.44
	Inferior parietal gyrus R	37.5	-54	52.5	4.00	3.61
22615	Medial frontal orbital gyrus R	7.5	57	-9	6.36	5.17
	Middle frontal gyrus L	-36	42	15	5.48	4.64
	Superior frontal orbital gyrus L	-28.5	52.5	-1.5	5.33	4.55
16081	Fusiformgyrus R	24	-40.5	-12	5.45	4.62
	Superior temporal gyrus R	55.5	-4.5	-1.5	5.28	4.52
	Hippocampus R	19.5	-13.5	-18	5.28	4.51
1295	Superior occipital gyrus L	-21	-70.5	37.5	5.30	4.53
	Angular gyrus L	-45	-63	24	4.09	3.68
	Middle occipital L	-31.5	-78	21	3.86	3.51
2883	Median cingulated cortex L	-6	-10.5	42	5.27	4.51
	Median cingulatecortex R	7.5	-16.5	45	4.96	4.30
533	Middle frontal orbital gyrus R	30	43.5	-15	5.05	4.36
227	Inferior parietal gyrus L	-39	-39	39	5.00	4.33
250	Superior	-51	-3	-10.5	4.39	3.90

	temporal gyrus L					
	Middle temporal gyrus L	-57	-15	-4.5	3.67	3.36
**AD atrophy**						
**Voxels per cluster**	**Regions**	**Coordinates**			**Peak t**	**Peak z**
		x	y	Z		
2155	Amygdala R	25.5	-4.5	-15	4.84	4.15
2420	Fusiformgyrus R	25.5	-42	-12	4.62	4.00
245	Superior frontal gyrus R	24	10.5	58.5	4.36	3.82
1004	HippocampusL	-27	-9	-21	4.21	3.72
215	Insula R	46.5	4.5	-6	4.10	3.64
764	Fusiformgyrus L	-37.5	-51	-13.5	3.96	3.54
72	Middle frontal gyrus L	-28.5	33	31.5	3.78	3.40

L: left, R: right

Supplementary Table 4. Correlations considering regions inside the masks.

	**bvFTD**		**AD**	
**Confidence regions**	**r**	***p* values FDR corrected**	**r**	***p* values FDR corrected**
Inferior frontal orbital gyrus R	-0.33	NS	-0.41	.03
Superior frontal orbital gyrus R	-0.32	NS	-0.42	.03
Olfactorygyrus R	-0.31	NS	-0.39	.03
Parahippocampus R	-0.28	NS	-0.35	.04
Inferior frontal orbital gyrus L	-0.39	NS	-0.41	.03
Superior frontal orbital gyrus L	-0.34	NS	-0.42	.03
Olfactorygyrus L	-0.33	NS	-0.41	.03
Parahippocampus L	-0.33	NS	-0.35	.04
**Wagering regions**				
Inferior frontal opercular gyrus R	-0.51	.002	-0.52	.002

Inferior frontal orbital gyrus R	-0.61	< .001	-0.65	< .001
Inferior frontal gyrus, pars triangularis R	-0.59	< .001	-0.59	< .001
Middle frontal gyrus R	-0.52	.002	-0.55	.001
Heschl R	-0.50	.002	-0.6	< .001
Insula R	-0.52	.002	-0.62	< .001
Postcentralgyrus R	-0.30	NS	-0.47	.006
Precentralgyrus R	-0.48	.004	-0.5	.003
Putamen R	-0.44	.006	-0.45	.008
Rolandic operculum R	-0.45	.005	-0.52	.002
Superior temporal pole R	-0.52	.002	-0.56	.001
Superior temporal gyrus R	-0.56	.002	-0.63	< .001
Inferior frontal opercular gyrus L	-0.44	.007	-0.5	.003
Inferior frontal orbital gyrus L	-0.50	.002	-0.64	< .001
Inferior frontal gyrus, pars triangularis L	-0.53	.002	-0.57	< .001
Middle frontal gyrus L	-0.54	.002	-0.62	< .001
Heschl's gyrus L	-0.46	.005	-0.66	< .001
Insula L	-0.49	.003	-0.59	< .001
Postcentralgyrus L	-0.35	.029	-0.45	.007
Precentralgyrus L	-0.46	.005	-0.53	.002
Putamen L	-0.41	.011	-0.47	.006
Rolandic operculum L	-0.42	.009	-0.5	.003
Superior temporal pole L	-0.36	.025	-0.41	.015
Superior temporal gyrus L	-0.46	.005	-0.66	< .001

L: left, R: right
NS: Non significant

3. REFERENCES

1 Rascovsky, K. *et al.* Sensitivity of revised diagnostic criteria for the behavioural variant of frontotemporal dementia. *Brain : a journal of neurology* **134**, 2456-2477, doi:10.1093/brain/awr179 (2011).

2 Ibanez, A. & Manes, F. Contextual social cognition and the behavioral variant of frontotemporal dementia. *Neurology* **78**, 1354-1362, doi:10.1212/WNL.0b013e3182518375 (2012).

3 Neary, D. *et al.* Frontotemporal lobar degeneration: a consensus on clinical diagnostic criteria. *Neurology* **51**, 1546-1554 (1998).

4 Piguet, O., Hornberger, M., Mioshi, E. & Hodges, J. R. Behavioural-variant frontotemporal dementia: diagnosis, clinical staging, and management. *The Lancet. Neurology* **10**, 162-172, doi:10.1016/S1474-4422(10)70299-4 (2011).

5 McKhann, G. M. *et al.* The diagnosis of dementia due to Alzheimer's disease: recommendations from the National Institute on Aging-Alzheimer's Association workgroups on diagnostic guidelines for Alzheimer's disease. *Alzheimer's & dementia : the journal of the Alzheimer's Association* **7**, 263-269, doi:10.1016/j.jalz.2011.03.005 (2011).

6 McKhann, G. *et al.* Clinical diagnosis of Alzheimer's disease: report of the NINCDS-ADRDA Work Group under the auspices of Department of Health and Human Services Task Force on Alzheimer's Disease. *Neurology* **34**, 939-944 (1984).

7 Baez, S. *et al.* Comparing moral judgments of patients with frontotemporal dementia and frontal stroke. *JAMA neurology* **71**, 1172-1176, doi:10.1001/jamaneurol.2014.347 (2014).

8 Torralva, T., Roca, M., Gleichgerrcht, E., Bekinschtein, T. & Manes, F. A neuropsychological battery to detect specific executive and social cognitive impairments in early frontotemporal dementia. *Brain : a journal of neurology* **132**, 1299-1309 (2009).

9 Sedeno, L. *et al.* Tackling variability: A multicenter study to provide a gold-standard network approach for frontotemporal dementia. *Human brain mapping* **38**, 3804-3822, doi:10.1002/hbm.23627 (2017).

10 Ashburner, J. A fast diffeomorphic image registration algorithm. *NeuroImage* **38**, 95-113, doi:10.1016/j.neuroimage.2007.07.007 (2007).

11 Good, C. D. *et al.* A voxel-based morphometric study of ageing in 465 normal adult human brains. *Neuroimage* **14**, 21-36, doi:10.1006/nimg.2001.0786 (2001).

12 Irish, M., Piguet, O., Hodges, J. R. & Hornberger, M. Common and unique gray matter correlates of episodic memory dysfunction in frontotemporal dementia and Alzheimer's disease. *Human brain mapping* **35**, 1422-1435, doi:10.1002/hbm.22263 (2014).

13 Melloni, M. *et al.* Your perspective and my benefit: multiple lesion models of self-other integration strategies during social bargaining. *Brain : a journal of neurology* **139**, 3022-3040, doi:10.1093/brain/aww231 (2016).

14 García-Cordero, I. *et al.* Feeling, learning from and being aware of inner states: interoceptive dimensions in neurodegeneration and stroke. *Philosophical Transactions of the Royal Society B: Biological Sciences* **371** (2016).
